# The link between SARS-CoV-2 related microglial reactivity and astrocyte pathology in the inferior olivary nucleus

**DOI:** 10.3389/fnins.2023.1198219

**Published:** 2023-06-28

**Authors:** Nacoya Madden, Ying Zi Jessy Mei, Kelly Jakubiak, Juncheng Li, Gunnar Hargus, James E. Goldman, Osama Al-Dalahmah

**Affiliations:** Department of Pathology and Cell Biology, Columbia University Irving Medical Center, New York Presbyterian Hospital, New York, NY, United States

**Keywords:** astrocyte, microglia, inferior olivary nucleus, COVID-19, hypoxia

## Abstract

The pathological involvement of the central nervous system in SARS-CoV2 (COVID-19) patients is established. The burden of pathology is most pronounced in the brain stem including the medulla oblongata. Hypoxic/ischemic damage is the most frequent neuropathologic abnormality. Other neuropathologic features include neuronophagia, microglial nodules, and hallmarks of neurodegenerative diseases: astrogliosis and microglial reactivity. It is still unknown if these pathologies are secondary to hypoxia versus a combination of inflammatory response combined with hypoxia. It is also unknown how astrocytes react to neuroinflammation in COVID-19, especially considering evidence supporting the neurotoxicity of certain astrocytic phenotypes. This study aims to define the link between astrocytic and microglial pathology in COVID-19 victims in the inferior olivary nucleus, which is one of the most severely affected brain regions in COVID-19, and establish whether COVID-19 pathology is driven by hypoxic damage. Here, we conducted neuropathologic assessments and multiplex-immunofluorescence studies on the medulla oblongata of 18 COVID-19, 10 pre-pandemic patients who died of acute respiratory distress syndrome (ARDS), and 7–8 control patients with no ARDS or COVID-19. The comparison of ARDS and COVID-19 allows us to identify whether the pathology in COVID-19 can be explained by hypoxia alone, which is common to both conditions. Our results showed increased olivary astrogliosis in ARDS and COVID-19. However, microglial density and microglial reactivity were increased only in COVID-19, in a region-specific manner. Also, olivary hilar astrocytes increased YKL-40 (CHI3L1) in COVID-19, but to a lesser extent than ARDS astrocytes. COVID-19 astrocytes also showed lower levels of Aquaporin-4 (AQP4), and Metallothionein-3 in subsets of COVID-19 brain regions. Cluster analysis on immunohistochemical attributes of astrocytes and microglia identified ARDS and COVID-19 clusters with correlations to clinical history and disease course. Our results indicate that olivary glial pathology and neuroinflammation in the COVID-19 cannot be explained solely by hypoxia and suggest that failure of astrocytes to upregulate the anti-inflammatory YKL-40 may contribute to the neuroinflammation. Notwithstanding the limitations of retrospective studies in establishing causality, our experimental design cannot adequately control for factors external to our design. Perturbative studies are needed to confirm the role of the above-described astrocytic phenotypes in neuroinflammation.

## Introduction

COVID-19, an infection caused by the coronavirus SARS-CoV-2, can lead to an acute severe respiratory syndrome that has caused millions of deaths in recent years Patients with COVID-19 exhibit respiratory symptoms severe enough to cause acute respiratory distress syndrome (ARDS) requiring hospitalization and usually mechanical ventilation (Aiyegbusi et al., 2021; Swenson and Swenson, 2021). The pathologic counterpart of ARDS is known as diffuse alveolar damage (DAD) (Konopka et al., 2020), a condition that leads to alveolar damage and failure of gas exchange, culminating in hypoxemia (Swenson and Swenson, 2021). The brain is particularly vulnerable to hypoxemia and COVID-19 patients are known to exhibit acute and chronic neurologic symptoms and sequelae (Mao et al., 2020; von Weyhern et al., 2020). We and several other groups have conducted neuropathologic studies to determine the neuropathologic features of COVID-19 in the brain (al-Dalahmah et al., 2020a; Deigendesch et al., 2020; Matschke et al., 2020; Solomon et al., 2020; Colombo et al., 2021; Fabbri et al., 2021; Thakur et al., 2021; Wierzba-Bobrowicz et al., 2021; Agrawal et al., 2022). The main neuropathologic findings across multiple datasets point to ischemia, hemorrhage, astrogliosis and microgliosis as the primary neuropathologic insults, with very little evidence to support direct invasion of the brain by the virus (Maiese et al., 2021). Given that these neuropathologic insults are non-specific, and can be seen in, and therefore explained by, brain hypoxia, we designed this study to directly address this question: Can the neuropathologic findings in COVID-19 be explained by hypoxic injury alone?

Astrogliosis and microgliosis are salient to neurodegeneration and neuroinflammation (Kwon and Koh, 2020; Muzio et al., 2021; Vandenbark et al., 2021). In COVID-19 brains, astrogliosis and microgliosis are common (Maiese et al., 2021). Astrogliosis is characterized by morphologic and functional alterations secondary to pathologic tissue damage and can lead to a combination of changes in homeostatic, neuroprotective, and/or neurotoxic functions (Escartin et al., 2021). Usually, reactive astrocytes exhibit increased GFAP levels associated with hypertrophy and/or proliferation (Sofroniew and Vinters, 2010). Likewise, microgliosis or microglial reactivity is associated with morphologic and functional alterations secondary to pathologic insults and tissue damage. This is usually associated with morphologic changes including loss of the ramified appearance and retraction of cell processes, and increased expression of activation molecules like MHCII proteins and CD68 (Woodburn et al., 2021). Astrogliosis and microgliosis may be secondary to tissue damage, but can also adopt central roles in neurodegeneration. For instance, mutations that impair microglial function such as those involving TREM2 are associated with increased risk of Alzheimer’s disease (Hansen et al., 2018). Importantly, the cross-talk between microglia and astrocytes is an actively researched topic in glial biology and neurodegeneration (Matejuk and Ransohoff, 2020). Microglia can drive astrogliosis (Liddelow et al., 2017), and astrocytes can regulate microglial reactivity (Cekanaviciute and Buckwalter, 2016; Chhatbar et al., 2018).

We are interested in the phenotypes of astrocytes in COVID-19 brains. We chose to study reactive astrocytes by performing detailed immunohistochemical analyses of astrocyte protein expression in the inferior olivary nucleus (ION), which is one of the most commonly and severely affected regions in COVID-19 brains (al-Dalahmah et al., 2020a; Thakur et al., 2021). The inferior olivary nuclei are located bilaterally within the rostral part of the medulla oblongata and participate in motor learning and coordination. ION neurons project via the hilum to contralateral cerebellar Purkinje cells (Schweighofer et al., 2013). In this study, we used post-mortem human tissue from control subjects who died with no neuropathologic abnormalities (*n* = 7–8), patients who died with ARDS before the COVID-19 pandemic (*n* = 10), and subjects who died from COVID-19 (*n* = 19). We performed immunohistochemistry and multiplex immunofluorescence studies for markers and microglia. We first established that ARDS and COVID-19 patients exhibited increased astrogliosis compared to controls. Because both ARDS and COVID- 19 patients had similar clinical courses, with profound hypoxia, in most cases requiring intubation, and the main difference between the two groups is the presence or absence of COVID-19 infection, we focused on these groups to investigate microglial reactivity and astrocyte protein expression. The ventral, lateral and dorsal regions of the ION along with the hilum were analyzed to examine the differences in microglial reactivity and astrocyte protein expression between ARDS and COVID-19 patients. We further employed principal component analysis and clustering methods to correlate astrocyte protein expression to the clinicopathologic attributes of the patients. Our findings represent one of the first attempts to address the question of whether neuropathology in COVID-19 is due to hypoxia alone vs. other factors, and link astrocyte protein expression to an exaggerated microglial response in the ION.

## Materials and methods

### Human brain samples

This study is in compliance with the Declaration of Helsinki. Consent for autopsy was obtained from the patient’s next of kin through standardized consenting procedures. No IRB approval was required given that the autopsy material used herein is considered to be non-human subjects. Pre-COVID autopsy material was obtained from donors who died between January 2018–2019, or during 2020–2021 and were negative for COVID-19. COVID-19 cases are previously thoroughly described (al-Dalahmah et al., 2020a; Thakur et al., 2021). Only the medulla oblongata tissue was analyzed in this study. The demographic information and relevant information regarding hospital course, including whether patients had histologic evidence of Diffuse Alveolar Damage (DAD) in the case when a full autopsy had been conducted, are provided in Supplementary Table S1. For some of our control cases, the clinical history is not available. This is because these were brain-only autopsies of patients who died elsewhere (not in our hospital). For these cases, we ensured that no hypoxic changes were neuropathologically detected (i.e., red neurons, nuclear pyknosis, and neuronal shrinkage) so as to use them as non-hypoxic controls. Autopsy brains, fixed in 10% formalin for 10–14 days after removal, were sectioned coronally and samples from representative areas of the CNS were removed and embedded in paraffin blocks, cut at 7 μm thickness, and mounted on charged glass slides. All studies reported herein are from the medulla at the level of the inferior olivary nuclei and hypoglossal nuclei.

### Immunohistochemistry

All immunostains were conducted on a Leica^©^ Bond RXm automated stainer. For chromogenic 3,3′-Diaminobenzidine (DAB) stains, a generic immunohistochemistry protocol was employed as per manufacturer protocols. For multiplexing immunostains using antibodies raised in non-overlapping hosts, we used a generic immunofluorescence protocol. Briefly, slides were baked in a 65°C oven for a minimum of 2 h. The following protocol was then used: After a dewaxing step, incubation in BOND Epitope Retrieval Solution 2 (cat# AR9640) for 20 min was used for heat-induced epitope retrieval. Next, the slides were washed in 1X PBS before washing twice in Bond Wash Solution (Ref#AR9590)—10 min/wash. Next, they were incubated in a 10% donkey serum blocking buffer for 60 min followed by the primary antibody diluted in blocking buffer for 60 min. After three washes, the slides were incubated in the secondary antibody containing buffer for 60 min. After three washes, A DAPI containing mounting solution (Everbright TrueBlack Hardset Mounting Medium with DAPI Cat#23018) was used to label nuclei and quench autofluorescence prior to coverslipping. One hundred fifty microliters/slide was the volume we used for all steps. All steps were conducted at ambient temperature—excluding the antigen retrieval step.

For multiplexing immunostains using primary antibodies raised in overlapping hosts (ALDH1L1, MT3 and AQP4 and ALDH1L1, YKL-40 and C3), the Opal 4-color Automation IHC kit Ref#220126024 from Akoya^©^ Biosciences was used in accordance with the manufacturer protocol. Briefly, two wash steps were followed by incubation in PKI Blocking buffer for 5 min before incubation in the first primary antibody for 30 min. After 3 wash steps, the slides were incubated in Opal Polymer HRP for 10 min followed by 6 wash steps prior to incubation in Opal 520 reagent for 10 min. This was followed by 4 additional wash steps. Next, the slides were incubated in Bond ER 1 solution for 20 min at 95° to elute the antibody complexes before 3 more wash steps. This procedure was repeated twice, once with the second primary antibody and Opal 570 reagent and once with the third primary antibody and Opal 690 reagent. Following the 3 wash steps at the end of the third round, the slides were incubated with Spectral DAPI for 5 min before the final 3 wash steps.

The following primary antibodies and dilutions were used: Rabbit ALDH1L1 (1:100, EnCor, Cat#RPCA-ALDH1L1), Rabbit YKL-40 (1:250, Abcam, Cat#ab255297), Rabbit C3 (1:200, Abcam, Cat#ab200999), Chicken GFAP (1:1000, Abcam, Cat#4674), Goat Clusterin (1:200, Thermo fisher, PA5-46931), Rabbit CD44 (1:100, Abcam, Cat#ab101531), Rabbit MT3 (1:100, millipore, Cat#HPA004011), Rabbit, AQP4 (1:2000, Millipore, Cat# ABN910), Goat IBA1 (1,500, Abcam, Cat#ab5076), Rabbit Trem2 (1,100, Cell Signaling, Cat#91068). Secondary antibodies conjugated to fluorophores: anti-mouse Alexa Fluor 488, 568, and 633, anti-rabbit Alexa Fluor 488, 594, anti-chicken Alexa Fluor 488 and 647, and anti-goat Alexa Fluor 488, 568, 633; all from goat or donkey (1:500, ThermoFisher Scientific, Eugene, OR).

### Imaging

All brightfield images were taken using a Leica Aperio LSM^™^ slide scanner under 20X objective. All immunofluorescent images were taken on the Leica Thunder imager DMi8. Images were acquired at 20x using a Leica K5 camera. Leica biosystems LAS X software was used for image capture. Tiles covering the entire ION were taken and stitched. Leica Thunder instant computational clearing was used to remove out of focus light. The images were exported as tiff files for downstream analysis.

### Image analysis

All image analysis was done in QuPath 0.30 (Bankhead et al., 2017). Annotations delineating the ventral, lateral and dorsal ION Parenchyma as well as the hilum were manually drawn. To detect cells, we used the “cell detection” function under the analysis menu. The DAPI Channel was selected for the Detection Channel. We modified the background threshold for each image to eliminate non-specific detections. Next, we trained an object classifier to classify the detections for the different channels. Training data were created from each image to delineate cells that are positive for the specific antigens in question. One classifier per channel was trained by calling the “train object classifier” function under classify with the following parameters: type = Random Trees, measurements = Cell: measurements = Cell: Channel X standard deviation, mean, max, and min measurements for the channel in question. To increase the accuracy of the classifier, additional training annotations were created on the image in question until the classification results matched the impression of the observer. Once a classifier was trained for each channel, “create composite classifier” was called to create a classifier consisting of multiple individual classifiers, one for each channel on the image. Classifiers were trained for each image separately. For CD44 and AQP4 analysis, we created a pixel classifier to classify positive and negative pixels. Training annotations were created for each image for positive and negative pixels. “Train pixel classifier” function was then called with the classifier type set to random trees, with a resolution of 2.60 μm/pixel, and selected all the features from only the channel in question.

To measure the minimal distance between microglial and olivary neurons, we first detected microglia using positive cell detection to identify IBA1+ cells. Next, QuPath pixel classifier was used to classify IBA1− ION neurons, which have characteristic large cytoplasm and eccentric nuclei. Using more than 20 manually annotated neurons as the training set, the pixel classifier accurately detected all neurons. We next converted the pixel classifications into annotations which we used to measure the distance against by calling analyze > spatial analysis > distance to annotations 2D measurements function between microglia (as positive cells) and neurons (as annotations). The measurements were exported as .csv files for downstream analysis in R. After −1*log10(1 + value) normalization, and binning into 100 bins, the kernel density distribution of the counts of cells that fall within each bin was used to calculate the modes for each condition using the multimode package in R by calling the locmodes function with the following options (lowsup = 0.00001, uppsup = 6, mod0 = 2, display = T). The supports were chosen to fit the data empirically—the upper support was ≤ to the maximum value in the data. We assumed two modes for the distribution (mod0 = 2). The Gaussian kernel density estimator is employed in the package. The distributions were compared using the ks test (two-sided) in R.

To classify microglia by activation state, an object classifier was used on objects detected by setting the detection channel to the IBA1 channel. This allowed the full tracing of microglial processes. The training images for microglial reactivity were compiled from examples taken from all images included in the analysis. Training objects were assigned by setting the class of microglia as quiescent vs. activated cells. The key characteristics used to identify a quiescent microglial cell were lightly-stained processes and small somata, while the activated microglia were marked by darker stains, larger soma, and thickened and retracted processes.

### PCA and cluster analysis

PCA analysis was done in FactoMineR R package (Lê et al., 2008). A total of 28 donor brains (10 ARDS and 18 COVID) were analyzed in four brain regions (dorsal, lateral, and ventral ION parenchyma (OP), and hilum), for a total of 107 data points representing the results of the image quantification after outlier removal. Metadata was included in the analysis as supplementary variables. Numerical values (age and length of hospitalization) were categorized into three bins. Other qualitative data included presence or absence of diffuse alveolar damage (DAD), intubation, and sepsis, as well as sex, condition, and brain region. −1*Log10 (value+1) normalization was performed on all immunohistochemical data measured as number of positive cells per area; no normalization was performed for data measured by percentage of area covered (AQP4 and CD44). Outliers, denoted in Supplementary Table S1, were identified in both using the Grubb’s method (see Section Statistical Analysis section below) and in cluster analysis. Outliers formed small 1–2 sample clusters. The few missing values, such as those resulting from low quality images, were imputed using the imputePCA function of the MissMDA package in R. Principal component analysis (PCA) was performed on the IHC data alongside supplementary qualitative variables, comprised of the metadata variables (Supplementary Figure S5A). The dimdesc function, part of the FactoMineR package, provided further details of factor analysis of samples (Supplementary Table S2). These results were then used for hierarchical clustering analysis, using the FactoMineR package’s HCPC function with the distance metric set to ‘Manhattan’, to provide four hierarchical clusters of the data (Figure 5B). Proportions of qualitative variables comprising each hierarchical cluster were then calculated using Dplyr functions (Supplementary Figure S5C). Heatmaps were generated using the pheatmap R package.

### Statistical analysis

All statistical analyses were conducted in GraphPad^®^ Prism 9 or R v4.03. For all data sets, outliers were identified using the Grubbs’ method with an Alpha = 0.2. All statistical tests and graphs were done using the outlier-free data. For analyzing two groups we used two-tailed and unpaired *t*-tests. For analyzing more than two groups, we used one way Brown-Forsythe and Welch ANOVA correcting for multiple comparisons using Original FDR method of Benjiamini and Hochberg. All data sets that were analyzed using one-way ANOVA were tested for normality using Shapiro–Wilk test and transformed using Y = −1*Log(Y + 1) if they did not pass the normality test. *p* values reported are those of the transformed data where transformation was done. To further validate our ANOVA test results, a beta regression model was also used as implemented in the betareg package in R. The independent variables used were condition (either ARDS, COVID with microglial nodules (MN), and COVID with no MN, or Control, ARDS, and COVID) and region (dorsal, lateral, and ventral OP), with the counts (microglia per area, proportion of activated microglia, GFAP per area, or percent MT3) as the dependent variable. The results of this analysis are provided in Supplementary Table S4.

## Results

### Increased microglial activation in the ION of COVID patients

**Figure 1:**
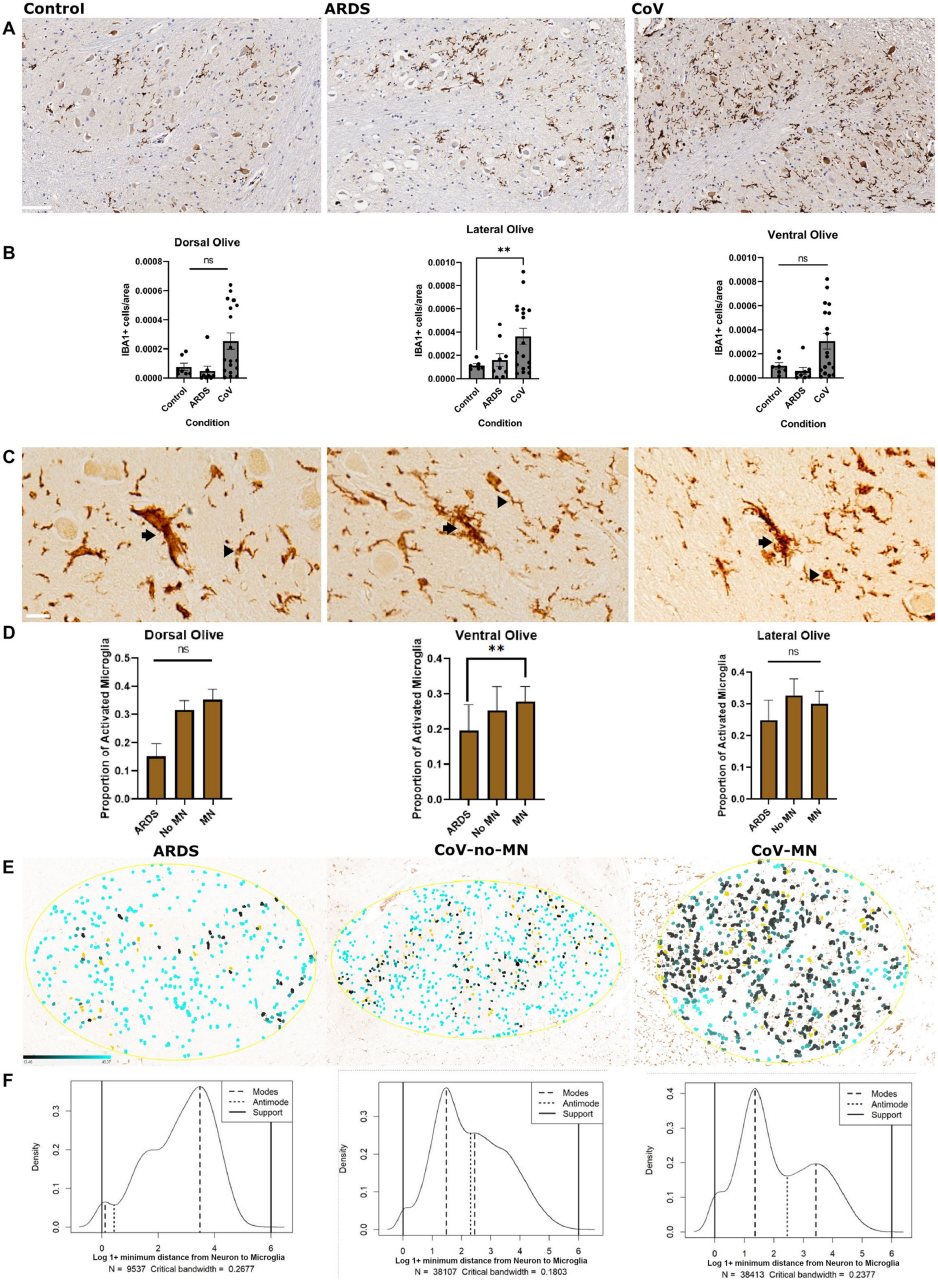
Microglial reactivity in COVID-19. **(A)** Immunohistochemical stain for IBA1 to label microglia in the ventral ION. Scale bar is 100 μm. **(B)** Quantification of the number of IBA1+ microglia per unit area in the dorsal, ventral and lateral ION. *N*= 18 for COVID-19, 10 for ARDS, and 7 for Control. The data was transformed using Y=-1*Log(Y) prior to calculating *P* values. ANOVA p-value= dorsal:0.0098, lateral: 0.0416, and ventral: 0.0076 based on transformed data. The graphs of transformed data are provided in table S4. *P* values of multiple comparisons: Control vs. CoV: dorsal: 0.1547, lateral: 0.0089 and ventral: 0.1372.Control vs ARDS: dorsal: 0.0582, lateral: 0.6543, and ventral: 0.0858.**(C)** Examples of different microglia classified as activated (arrow) versus quiescent (arrowhead). Scale bar is 20 μm. **(D)** Quantification of the percentage of total microglia that were classified as activated in each anatomic region (dorsal, ventral, and lateral). *N*= 10 for COVID-19 MN, 8 for COVID-19 No-MN, and 10 for ARDS. Normality was confirmed using a Shapiro-Wilk test. ANOVA *P*-values= dorsal: 0.84236, lateral: 0.1995, and ventral: 0.0174. Multiple comparisons *P* values = Control vs no-MN: dorsal:,0.0853, lateral: 0.2590 and ventral: 0.1973. Control vs CoV-MN: dorsal: 0.5654, lateral: 0.1551 and ventral: 0.0050. **(E)** Distance maps depicting the distance between neurons (masked in Yellow) and microglia in the Ventral Olive. The distance is shown as a color gradient (Black: close, cyan: far). The gradient is shown in the bottom left part of the left panel. **(F)** Probability density plots showing the probability distributions of the proportion of microglia that fall within a specified distance from the closest neuron, binned into 100 bins after log normalization. The modes (peaks) and anti-modes (troughs) are indicated. The supports indicate the upper and lower bounds of the distributions. The condition depicted in each graph is as in panel **E**. ARDS: Acute respiratory distress syndrome. MN: COVID-19 with microglial nodules. No-MN: COVID-19 without microglial nodules. **B** One way Brown-Forsythe and Welch ANOVA. Comparisons are against Control. **D** One way Brown-Forsythe and Welch ANOVA. Comparisons are against ARDS. Data is shown as mean +/- SEM.

Microglial reactivity is a common feature of COVID-19 pathology. We first set out to replicate this finding in our cohort, focusing on the ION. We included control patients who died without COVID-19 or ARDS, patients who died with ARDS but not COVID-19, and patients who died of COVID-19. This allows us to answer the following question: is microglial reactivity in COVID-19 due to hypoxia? Thus, we quantified the number of IBA1+ cells per unit area in different regions of the ION: the lateral, dorsal, and ventral sectors (Figure 1A). ANOVA analysis of IBA1+ cells/area was significant in all three regions of the ION, and there was a significant increase in the number of microglia per unit area in COVID-19 cases compared to the non-hypoxic controls in the lateral ION, but there was no difference between the non-hypoxic controls and the ARDS cohort (Figure 1B). This indicates that factors in COVID-19, in addition to hypoxia, were necessary to drive the increase in microglia in the ION. Beta regression analysis for microglia per area returned, for the COVID condition, a coefficient of 0.472 and *p* value 2.89E-05 (Supplementary Table S4), suggesting that COVID-19 condition can explain the increased microglial numbers in the COVID-19 cases in our cohort. Because ARDS and controls were not significantly different in the density of ION microglia, and ARDS cases can be considered matching controls for hypoxia, we compared microglial reactivity between the ARDS and COVID-19. We used morphologic attributes of microglia to train a machine learning algorithm to classify microglia into quiescent versus activated (see Section Materials and methods). We wanted a simple way to classify microglia based on morphology, knowing that microglial reactivity falls on a spectrum of states, and that activated microglia generally have retracted thick processes compared to quiescent cells (Davis et al., 2017; Leyh et al., 2021). We opted for a simple binary classification of quiescent vs. activated microglia; we show examples of these classifications in Figure 1C. We also chose to split our COVID-19 group into two groups: a group with high abundance of microglial nodules (MN), and another group with relatively few/no microglial nodules (No-MN). These designations were based on previously reported neuropathologic assessments (Thakur et al., 2021). Comparing the proportion of activated microglia across the ventral, dorsal, and lateral ION in these three groups (ARDS, COVID-19 no-MN, COVID-19 MN) revealed that in the ventral ION, the COVID-19 MN group had a significantly larger proportion of activated microglia (Figure 1D). Subsequent beta regression testing of proportion of activated microglia returned a coefficient of 0.509 with *p* value 0.000553 for the COVID-MN condition, but the coefficients were not significant for ION regions, suggesting that condition rather than ION region drives microglial activation (Supplementary Table S4). Next, we asked if the minimum distance between any microglial cell and the closest neuron to it is different between the groups. This in effect is a way to quantify the proximity of microglia to neurons. We reasoned that we would see more microglia close to neurons if there is more neuronophagia or microglial nodules. We measured the distance between microglia and neurons in the ION (Figure 1E) and found that the distribution of minimal distance between microglia and neurons is quite different between the groups (Figure 1F—Asymptotic two-sample Kolmogorov–Smirnov test—a non-parametric test to compare distributions). The results are as follows: *D* values = 0.28, 0.21, 0.12, for MN vs. ARDS, No-MN vs. ARDS, and MN vs. No-MN, respectively, for all comparisons the *p*-value is less than 2.2e-16. Comparing the distribution across different anatomic sectors of the ION revealed similar results (data not shown). The distribution was truly bimodal in the COVID-19 MN group, and the two modes were 1.36 (higher probability mode) and 3.45 (lower probability mode). Conversely, the modes for the ARDS group were 3.47 (highest probability mode) and 0.11 (lower probability mode) and for the No-MN group were 1.48 (highest probability mode) and 2.45 (lower probability mode). The fact that the mode with the highest probability (density) in the MN was lower than that in the ARDS group can be seen as an indirect measure of the presence of microglial nodules in the MN group—which is previously established. Altogether, we found that microglia are more activated and closer to neurons in the ION in COVID-19, especially the MN group.

We also asked if microglia in COVID-19 brains expressed more TREM2 compared to microglia in ARDS in the ION. TREM2 labels phagocytic microglia (Takahashi et al., 2005). Although we could detect TREM2 in microglia in the white matter surrounding the ION (for example—in the pyramids Supplementary Figure S1A), there was no significant specific labeling of microglia in the ION in COVID-19 or in ARDS (Supplementary Figure S1B).

### Increased astrogliosis in ARDS and COVID-19 patients

**Figure 2:**
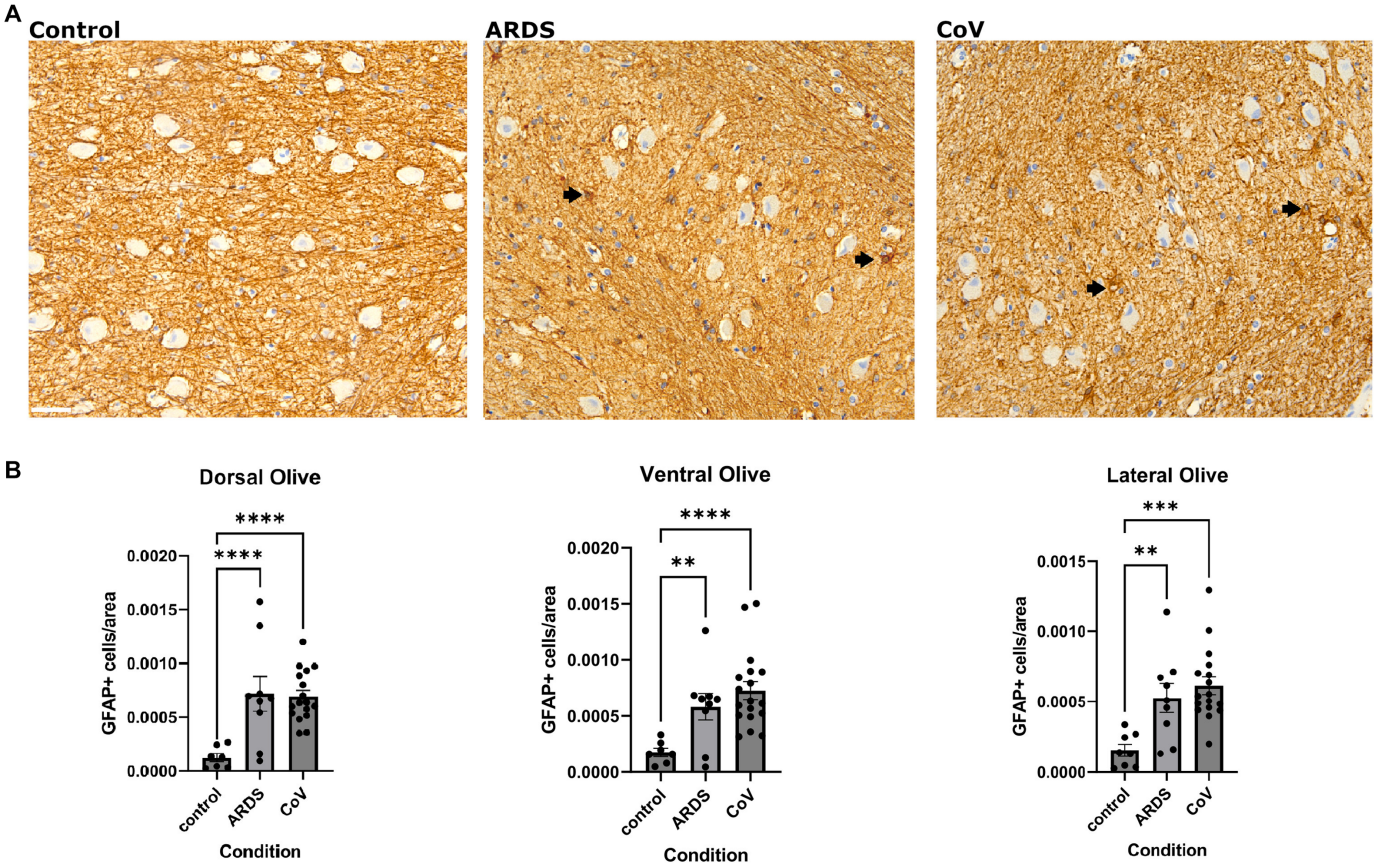
Astrogliosis in COVID-19. **(A)** Immunohistochemical stain for GFAP in the ventral ION. Black arrows point to GFAP positive cells. Scale bar indicates 50 μm. **(B)** Quantification of GFAP positive cells per unit area in the non-hypoxic controls, ARDS and COVID-19 samples across the dorsal, lateral, and ventral regions of the ION. One way Brown-Forsythe and Welch ANOVA correcting for multiple comparisons using Original FDR method of Benjiamini and Hochberg. Comparisons are against for COVID-19 and ARDS are both against Control. *N*= 18 for COVID-19, 10 for ARDS, and 7 for Control. Data is shown as mean +/- SEM. The data was transformed using Y=-1*log10(Y) before calculating p-values. ANOVA *P* value= dorsal: <.0001, lateral: <0.0001 and ventral: 0.0002. Multiple comparison *P*=values:<0.0001 for ARDS and CoV in dorsal, 0.0002 for ARDS and <0.0001 for CoV in the lateral and 0.0049 for ARDS and <0.0001 for CoV in the ventral.

Given that astrogliosis is a prominent feature of COVID-19 neuropathology, we asked if we could recapitulate this finding in the ION. To address this question, we conducted a series of immunohistochemical and multiplex immunofluorescence studies to quantify the expression of proteins related to reactive astrogliosis or alterations in astrocyte function. First, we quantified the number of Glial fibrillary acidic protein (GFAP) positive astrocytes in the ION in controls, ARDS, and COVID-19 (Figure 2A). Interestingly, we found that compared to control, both ARDS and COVID-19 exhibited increased numbers of GFAP+ astrocytes, defined as GFAP+ somata, per unit area in all ION regions (Figure 2B). Notably, while control samples showed many GFAP+ astrocytic processes, few astrocytic cell bodies were labeled. Additionally, beta regression testing of GFAP per area data returned coefficient −0.0828 and *p* value 2.1E-08 for the control condition, consistent with our ANOVA results (Supplementary Table S4).

A caveat is worth mentioning here: detecting increased GFAP+ cells does not necessarily suggest that there were more astrocytes in one group vs. the other. Some astrocytes may exhibit lower levels of GFAP below the sensitivity of the assay, and can upregulate GFAP in pathologic contexts allowing its detection. Either way, the downstream interpretation of this phenomenon supports that in hypoxia (ARDS and COVID-19), there are elevated levels of astrogliosis.

### Ventral ION astrocytes in COVID-19 show decreased Aquaporin-4 compared with ARDS

**Figure 3:**
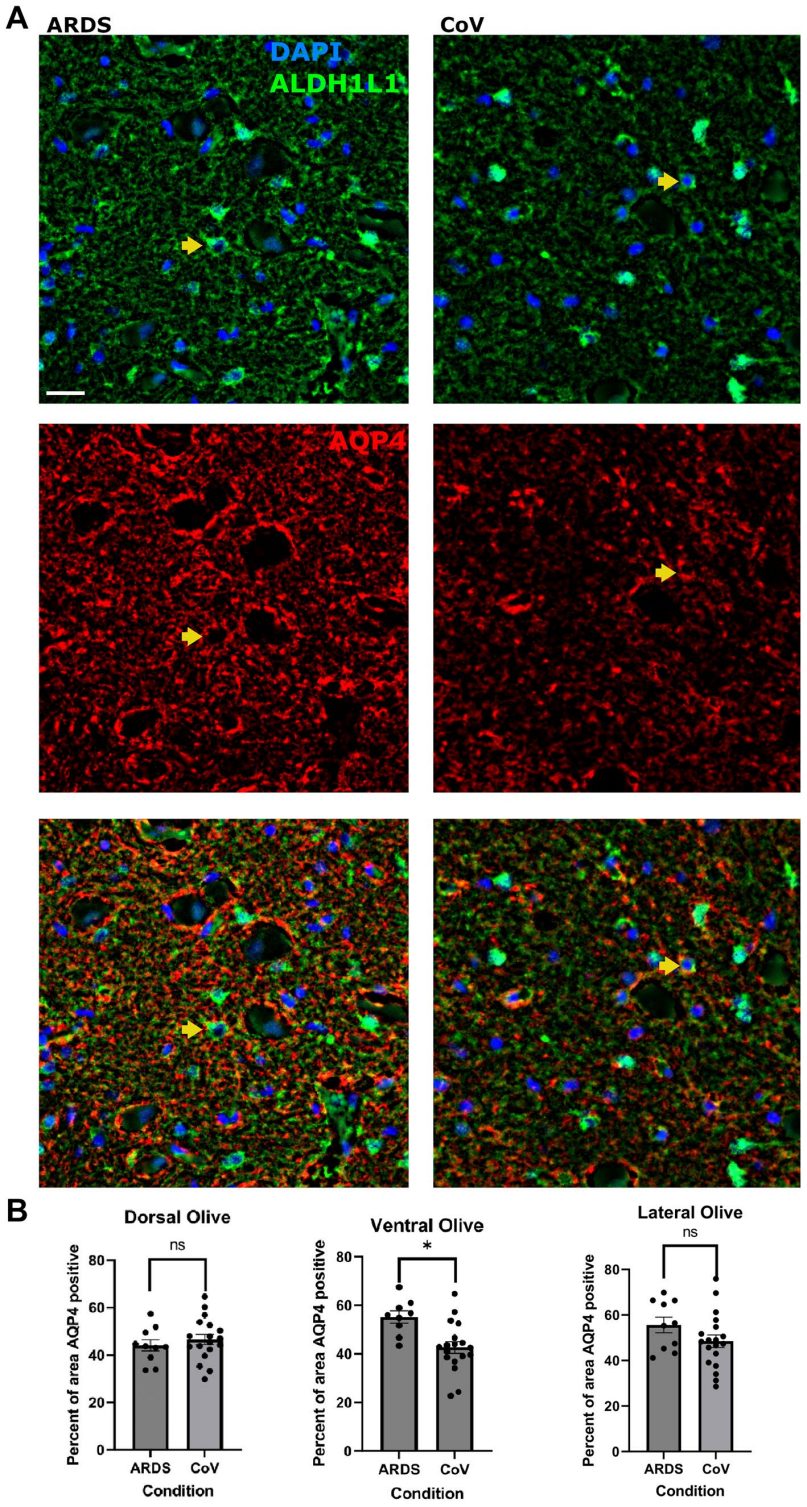
Lower AQP4 levels in the ventral ION in COVID-19 compared to ARDS. **(A)** Multiplex immunofluorescence showing ARDS (left) and COVID-19 (right) in the ventral ION labeled for nuclei (DAPI - blue) and ALDH1L1 (green - top panel), AQP4 (red – middle panel), and merged panels (lower panels). Arrows indicate cells positive for DAPI, ALDH1L1 and AQP4. Scale bar = 20 μm. **(B)** Quantification of the percent area positive for AQP4 per ION region. Unpaired two-tailed t-test. N= 18 for COVID-19, 10 for ARDS. *P* value = 0.0035. Data is shown as mean +/- SEM.

Reactive astrocytes upregulate the expression of Aquaporin-4 (AQP4) (Tomas-Camardiel et al., 2004; Tourdias et al., 2011), and redistribute its expression to the cell soma from the astrocytic end-feet, where it is normally localized (Eid et al., 2005; Steiner et al., 2012). Moreover, AQP4 has important implications in hypoxic–ischemic conditions (Shi et al., 2012), and studies have shown that loss of AQP4 protects against early cytotoxic edema associated with stroke (Papadopoulos and Verkman, 2008). Also, AQP4 expression in astrocytes has important implications in neuroinflammation secondary to ischemia, and AQP4 knockout mice exhibit exaggerated post-stroke microglial reactivity (Shi et al., 2012). Thus, we asked if AQP4 levels were altered in COVID-19 vs. ARDS. We measured the area covered by AQP4 in ION (Figure 3A). In patients who had died of COVID-19, there was decreased expression of AQP4 in the ventral ION compared to the ARDS patients (Figure 3B). Together, these findings link lower AQP4 levels to increase neuroinflammation in COVID-19.

### Hilar astrocytes of COVID-19 donors exhibit reduced levels of YKL-40 compared with ARDS

**Figure 4:**
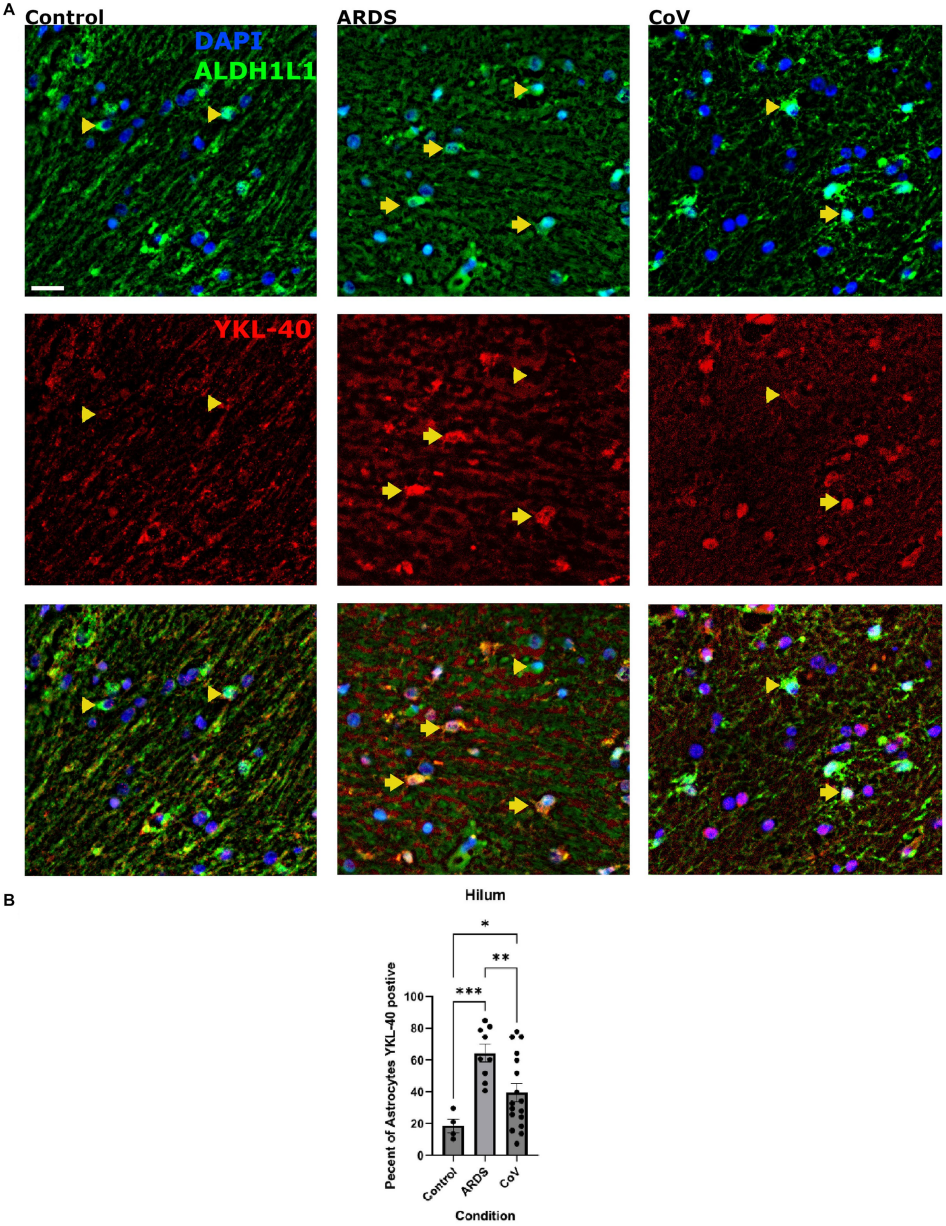
YKL-40 expression in the hila of the Controls, COVID-19 and ARDS. **(A)** Cells in the hilum stained for DAPI (blue) to detect nuclei of all cells and ALDH1L1 (green) to detect astrocytes. Scale bar = 20 μm. The next row shows YKL-40 (red) alone with the last figure being the merge of all three. **(B)** Quantification of the proportion of astrocytes positive for YKL-40 positive astrocytes per unit area in the hilum of non-hypoxic control, ARDS and COVID-19 cases  per ION region. One way Brown-Forsythe and Welch ANOVA correcting for multiple comparisons using Original FDR method of Benjiamini and Hochberg. *N*= 17 for COVID-19, 10 for ARDS, and 4 for controls. ANOVA *P* value = >0.0001. *P* value =<0.0001 for ARDS and 0.0106 for COVID-19. *P*-value of CoV in comparison to ARDS is 0.0091. Data is shown as mean +/- SEM.

Encoded by the *CHI3L1* gene, Chitinase-3-like protein (YKL-40) is a secreted glycoprotein primarily expressed in astrocytes in the brain that is a common marker of neurodegeneration (Querol-Vilaseca et al., 2017; Lananna et al., 2020; Zhao et al., 2020; Ferrari-Souza et al., 2022). Astrocytes increase the expression of YKL-40 in several neurodegenerative diseases including AD, tauopathies, and prion disease (Bonneh-Barkay et al., 2010; Llorens et al., 2017; Querol-Vilaseca et al., 2017). *In vitro* studies showed that YKL-40 could be induced in astrocytes by macrophages (Bonneh-Barkay et al., 2012). A recent study showed that YKL-40 knockout mice exhibit reduced amyloid plaques and increased expression of CD68 in microglia in an AD model, suggesting that YKL-40 suppresses microglial reactivity (Lananna et al., 2020). Thus, we examined the expression of YKL-40 in astrocytes in our cohort (Figure 4A). We were interested in knowing whether hypoxia in general can increase YKL-40, so for this analysis, we included the non-ARDS controls. We quantified the proportion of ALDH1L1 positive astrocytes that were also positive for YKL-40 and found that there were significantly more YKL-40 positive astrocytes in the hilum of ARDS and COVID-19 brains compared to non-ARDS controls (Figure 4B). This was not the case in the ION parenchyma (data not shown). However, there were fewer YKL-40 positive astrocytes in the ION hilum of the COVID-19 cohort compared to ARDS (Figure 4B). We examined astrocytic protein expression in all the comparisons we conducted, and YKL-40 is the only protein that we found dysregulated in the hilum. This is interesting given that it is a secreted protein (Zhao et al., 2020). Together, these results indicate that ION astrocytes behave differently under hypoxia in the setting of COVID-19 systemic infection; they fail to upregulate YKL-40 to the same extent as in ARDS. A caveat is that YKL-40 is a secreted protein, and that changes in YKL-40 levels between COVID-19 and ARDS may reflect changes in secretion patterns.

### Other markers of astrogliosis

To further characterize astrogliosis in COVID-19 ION astrocytes, we performed multiplex immunofluorescence for other protein markers associated with reactive astrogliosis. We first quantified the expression of metallothionein-3 (MT3), a zinc-binding protein that has been shown to be upregulated in reactive astrocytes in Huntington disease (al-Dalahmah et al., 2020b). Metallothioneins are thought to be neuroprotective (Stankovic et al., 2007). Quantification of MT3 in different sectors of the ION of ARDS and COVID-19 showed no significant difference in the proportion of astrocytes that label with MT-3 (unpaired *t*-test, ARDS and COVID-19 mean ± SEM = 8.901 ± 2.731 and 14.27 ± 4.462, respectively, *p* value = 0.2262), however, when we stratified COVID-19 by the presence or absence of microglial nodules, we detected significantly lower proportions of lateral ION astrocytes in the COVID-19 with microglial nodules compared with ARDS patients (Supplementary Figures S2A,B). In accordance with this result, beta regression testing showed that COVID-MN condition had coefficient of—0.6191 and *p* value 0.0016, and that ION regions also had significant coefficients (Supplementary Table S4). We next asked if ION astrocytes in COVID-19 increase the expression of complement factor 3 (C3), which is a gene that is upregulated in and therefore a marker of putative neurotoxic “A1” astrocytes (Liddelow et al., 2017). We found no significant increase in the proportion of C3+ astrocytes in COVID-19 vs. ARDS (Supplementary Figures S3A,B). Finally, we examined the expression of CD44, an astrocyte protein expressed in white matter astrocytes [see our preprint (Al Dalahmah et al., 2023)], astrocytes around large vessels, interlaminar astrocytes, and a subset of cortical astrocytes (Sosunov et al., 2014), as well as Clusterin (CLU), which is increased in neurodegenerative astrocytes in AD (Wojtas et al., 2020; Chen et al., 2021). We quantified the area covered by CD44 and again found no significant increase in CD44 labeling in the COVID-19 ION (Supplementary Figures S4A,B). Likewise, we found no significant differences in the proportion of ION astrocytes that were CLU-positive between COVID-19 and ARDS (Supplementary Figures S4A,C).

### Astrocyte IHC profiles and microglial reactivity drive cohort clustering

**Figure 5:**
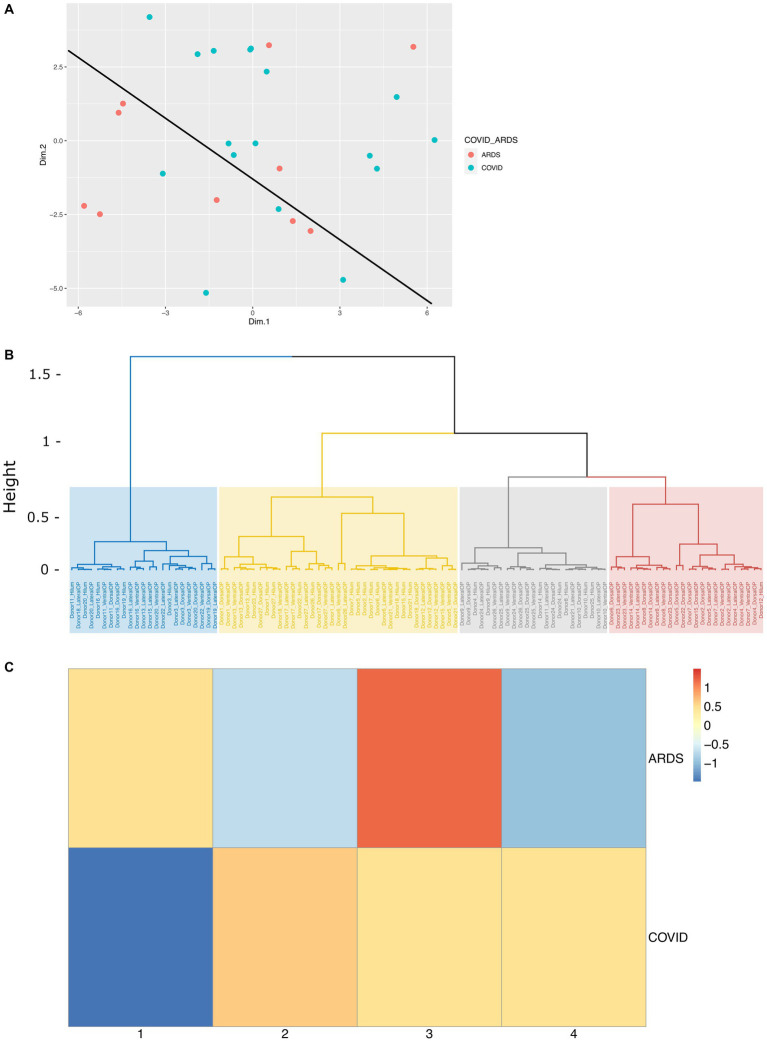
Cluster analysis of immunohistochemical data on COVID and ARDS cases. **(A)** PCA scatterplot of donors along PC1 and PC2, colored by condition (ARDS and COVID), which a line depicting the separation of COVID patients and ARDS patients in PCA space. **(B)** Dendrogram depicting the distribution of each sample into four hierarchical clusters. **(C)** Heatmap showing the proportion of all COVID samples and ARDS samples represented in each hierarchical cluster, scaled by row. Columns represent each of the four hierarchical clusters.

In our design, we tried to control for relevant demographic and clinical variables (metadata), however this is not always possible. To determine the correlation between metadata variables and biological results, we performed principal component analysis (PCA) on all the immunohistochemical data from images from different regions in ION, using the metadata as supplementary variables, allowing us to predict their PCA coordinates from the IHC data. The input to the PCA analysis is provided in Supplementary Table S2. First, we plotted the brain donors in PCA space and found that ARDS and COVID-19 donors were relatively well separated (Figure 5A). This highlights the biological differences between the two groups. A closer look at the PCA results showed that a number of quantitative IHC variables were responsible for the greatest amount of variation in dimensions 1 and 2 (Supplementary Figure S5; Supplementary Table S3). In PC1, YKL40, C3, and a combination of the two (YKL40.C3) had correlation values of 0.898, 0.839, and 0.910, and *p* values of 3.27E-39, 1.77E-29, and 4.68E-42, respectively. Interestingly, the proportion of activated microglia was only weakly correlated with PC1. Metadata variables length of hospital stay, condition, sex, and DAD had low correlation (R2 values of 0.206, 0.069, 0.039, and 0.038, respectively—Supplementary Table S3) with PC1, suggesting that our case-control matching is not perfect, but sufficient. CD44 proportion and CLU per area were the most significant IHC variables associated with PC2, with correlation values of 0.620 and 0.508, and *p* values of 1.09E-12 and 2.35E-08, respectively. Astrocytes per unit area and proportion of MT3 positive astrocytes were significantly and strongly negatively correlated with PC2. The most relevant qualitative variable for PC2 was age, with a relatively low R2 value of 0.080 (Supplementary Table S3). This again shows high significance but low correlation of qualitative variables with the variance shown in the dimension, suggesting that our ARDS- COVID-19 matching was relatively effective. Supplementary Figure S5A shows the correlation circle depicting the IHC variables and their correlation to PC1 and PC2. Supplementary Figure S5B shows the correlation between the metadata variables and the first 5 PCs.

We next asked if clustering the samples (IHC images) based on the PCA dimensions would give us clusters that reflect condition, and/or other relevant variables like anatomic region for example. To achieve that, we clustered the data on the first 5 PC’s using hierarchical clustering on the Euclidian distance matrix derived from the PC1-5 coordinates for each sample. We identified four clusters as shown in Figure 5B. Examination of the hierarchical clustering results show that clusters 1 and 4 were relatively deplete of samples derived from the ION hilum compared to clusters 2 and 3. This is expected because the hilum is composed of white matter harboring axons and glia, unlike the ION parenchyma, which harbors neurons, too. To highlight any relationships between clusters and condition (ARDS vs. COVID-19), we plotted the proportion of images that fell under each cluster against condition in a heatmap (Figure 5C). The results show that ARDS samples were mainly enriched in clusters 1 and 3, while COVID-19 samples were distributed between clusters 2, 3, and 4. Together, these findings demonstrate that our samples cluster based on the major factors that our analysis set out to investigate, biological condition and anatomic locale.

A closer look at the distribution of metadata variables shows cluster 1 appears to be most enriched with old age (13), and short hospital stay (16) samples, and cluster 2 with COVID (20), short hospital stay (22), male (19), DAD (20), and non-septic (23) samples. Cluster 3 is most enriched with median-age (20), ARDS (15), short hospital stay (22), DAD (22), and non-septic (26) samples, and cluster 4 with COVID (18) and female (16) samples (Supplementary Figure S2C). The cos2 value of each variable, a good metric of variable correlation with the circumference of the correlation circle, also shows the same patterns we described by looking at the R2 above. Briefly, correlations in PC1 with length of hospital stay, sex, DAD, disease condition including the presence of microglial nodules in COVID-19, in PC2 with region and age, and in PC3 with region and length of hospital stay, respectively (Supplementary Figure S2B). All together, these data suggest that in addition to condition and anatomic locale, sex, concomitant DAD, and length of hospital stay were variables that correlated with IHC features and contributed to clustering. However, their overall correlation with the PC’s that explain the variance was low, suggesting they had a modest influence on the reactivity of astrocytes and microglia in the ION.

## Discussion

This study investigated the effects of the SARS-CoV-2 (COVID-19) virus on astrocytes and microglia in the ION. We designed this study to control for hypoxemia by including controls with ARDS and no COVID-19 infection, allowing us to determine if systemic infection with SARS-CoV-2, independently drives glial pathology in the ION—one of the most severely involved brain regions in COVID-19 neuropathology (Thakur et al., 2021). We confirmed that our non-hypoxia controls had no neuropathologic evidence of hypoxia compared with the ARDS cases, which exhibited widespread hypoxic changes. We found that the ION in COVID-19 and ARDS exhibits significant astrogliosis, and in COVID-19 alone displays significant microgliosis. We found that COVID-19 microglia are closer to ION neurons compared with non-COVID-19 counterparts. We also found morphologic evidence for increased microglial reactivity in the ventral region of the ION. In parallel, we quantified astrocytic protein expression and found that in both COVID-19 and ARDS, YKL40 levels were increased in the hilum, however, the proportion of YKL-40+ hilar astrocytes was lower in COVID-19. Finally, ventral and lateral ION astrocytes in COVID-19 showed lower levels of AQP4 and MT3, respectively. Overall, our findings indicate that the pathology in COVID-19 cannot be explained by hypoxia alone, and that astrocytic pathology in COVID-19 may contribute to the prominent neuroinflammatory response in the brainstem.

Astrocytes play important roles in mediating the tissue response to hypoxia-ischemia (Vella et al., 2015), which is the most common neuropathologic abnormality in COVID-19 (Maiese et al., 2021). Astrocytes are primary drivers of cytotoxic edema in the acute phase of ischemia (Choi and Rothman, 1990; Pantoni et al., 1996; Nielsen et al., 1997), and vasogenic edema if the blood brain barrier breaks down (Badaut et al., 2002). AQP4 levels are increased in reactive conditions, and AQP4 can redistribute to the astrocytic somata during ischemia (Tourdias et al., 2011). Loss of AQP4 protects against early cytotoxic edema associated with stroke (Papadopoulos and Verkman, 2008). Therefore, it is possible that the reduction of AQP4 in the ventral ION in COVID-19 might be a protective response against ischemia. On the flip side, AQP4 knockout mice exhibit exaggerated post-stroke microglial reactivity (Shi et al., 2012), and this may explain the heightened microglial reactivity we see in COVID-19 ION. Perhaps this picture becomes more compelling when combined with the other phenotypic alterations we see in astrocytes, namely, the relative reduction of YKL-40, which is a secreted cytokine (Zhao et al., 2020) thought to suppress microglial reactivity (Lananna et al., 2020), and the relative failure of upregulation of the putative neuroprotective MT3. These findings along with those reported in this paper demonstrate the need for further mechanistic studies to investigate the functional roles of MT3, AQP4, and YKL-40 in astrocytes in animal or cell-based models. We did not find a gain of C3, which is a marker of putative neurotoxic “A1” astrocytes (Liddelow et al., 2017). Thus, it appears that COVID-19 astrocytes exhibit phenotypic alterations that may result in failure to check the immune response in the ION. Given that our controls were matched for hypoxemia, the alteration in astrocytic protein expression cannot be solely attributed to hypoxia. We can only conclude that some other factor, such as systemic infection with SARS-CoV-2, may underlie this astrocytic phenotype.

In considering potential causes for the astrocytic protein expression changes in the COVID-19 brains, we have to consider the role of comorbidities such as sepsis. We tried to control for this factor by patient matching, however, this is not always possible. Our PCA analysis indicates that sepsis is not significantly correlated with the first 2 PC’s, supporting that our patient matching approach was relatively effective at controlling for sepsis in this cohort. It has been shown that astrocytes in an animal model of lipopolysaccharide-induced sepsis increased expression of C3 (Zamanian et al., 2012; Liddelow et al., 2017). Had sepsis been the underlying reason behind astrocytic phenotypic changes, we would have detected changes in C3 expression, which was not the case. Another explanation for ION COVID-19 astrocyte phenotypes could be the increase in systemic levels of cytokines in COVID-19 (Chen et al., 2020). Unfortunately, we do not have data on the cytokine profiles from our cohort, and it would be impossible to retrieve that retrospectively from a postmortem dataset. Moreover, it is possible that systemic inflammation, as seen in COVID-19, may lead to alterations in the blood brain barrier (Varatharaj and Galea, 2017) which may then lead to changes in astrocyte phenotypes. Finally, we considered that astrocytes may be infected by SARS-CoV-2 directly leading to their phenotypic changes. To date, there is no convincing evidence that this happens in human tissue (al-Dalahmah et al., 2020a; Deigendesch et al., 2020; Matschke et al., 2020; Solomon et al., 2020; Colombo et al., 2021; Fabbri et al., 2021; Maiese et al., 2021; Thakur et al., 2021; Wierzba-Bobrowicz et al., 2021; Agrawal et al., 2022). Although we cannot rule it out completely, we conclude that direct infection of astrocytes by the virus given the available evidence is unlikely. We contend that systemic infection with SARS-CoV-2 indirectly alters astrocytic protein expression, and further studies are needed to examine this hypothesis.

There are notable limitations of this study. For starters, we examined astrocytic and microglial reactivity in only one region of the brainstem, the ION. We used this region as representative of the most severely affected brain regions acknowledging that there are other brain nuclei, like the dentate nucleus and the pontine nuclei, which also exhibit significant pathology in COVID-19 (al-Dalahmah et al., 2020a; Thakur et al., 2021). Future studies will examine these brain regions including others, to elaborate on the heterogeneous glial responses to injury in COVID-19. Another limitation is the incompleteness of the clinical data. It would have been optimal if the clinical records were complete so as to allow us to conduct more comprehensive analyses of the impact of several clinical variables on glial reactivity. We only included a limited number of variables for which we had data on most cases. We had to exclude our non-hypoxic controls from the analysis because our clinical records on these patients are lacking. These brain donors died elsewhere, outside the NY Presbyterian hospital, so we have no way of getting the relevant clinical information. Finally, our experimental design matched COVID-19 with ARDS patients for prolonged hypoxia, however, we cannot adequately control for other unmeasured factors that are beyond hypoxia and viral infection.

In conclusion, our data is the first to perform controlled immunophenotypic astrocytes in COVID-19 brains to determine whether the observed glial pathology can be explained by hypoxia. We found that hypoxia alone cannot explain glial pathology in COVID-19 in ION—one of the most severely affected regions in the brain. Future studies are needed to extend this approach to other brain regions that are severely affected vs. relatively preserved, to expand our understanding of the disease pathology. An unanswered question remains as to the regional heterogeneity of astrocytic and microglial reactivity in the ION, and further studies are needed to understand this phenomenon.

## Data availability statement

The original contributions presented in the study are included in the article/Supplementary material, further inquiries can be directed to the corresponding author.

## Ethics statement

Ethical review and approval was not required for the study on human participants in accordance with the local legislation and institutional requirements. The patients/participants provided their written informed consent to participate in this study.

## Author contributions

OA-D conceived and designed the study. NM, YM, KJ, and JL performed the immunostains. NM, YM, KJ, JL, GH, JG, and OA-D analyzed the data. NM, KJ, JG, and OA-D wrote the paper. All authors contributed to the article and approved the submitted version.

## Funding

OA-D was supported by the American Brain tumor Association—fully supported by the Uncle Kory Foundation and NIA ADRC REC program (Grant Number P30AG066462).

## Conflict of interest

The authors declare that the research was conducted in the absence of any commercial or financial relationships that could be construed as a potential conflict of interest.

## Publisher’s note

All claims expressed in this article are solely those of the authors and do not necessarily represent those of their affiliated organizations, or those of the publisher, the editors and the reviewers. Any product that may be evaluated in this article, or claim that may be made by its manufacturer, is not guaranteed or endorsed by the publisher.

## Supplementary material

SUPPLEMENTARY TABLE 1 Cohort demographic and clinical data.

SUPPLEMENTARY DATA SHEET 1 Data file containing the original set of immunohistochemical and meta data, following log10 normalization, outlier removal, and missing value imputation, used as input for all downstream analysis.

SUPPLEMENTARY DATA SHEET 2 PCA description of dimensions: Results from PCA analysis, giving details of the contribution of variables to dimensions 1 and 2, including only those variables with the greatest bearing on explanation of variance in those dimensions.

SUPPLEMENTARY DATA SHEET 3 Results from beta regression on proportions and percentage data implicated in ANOVA testing. Plots of transformed data where appropriate.

SUPPLEMENTARY FIGURE 1 TREM2 expression in ION microglia. **(A)** Immunohistochemical stains for TREM2 in a COVID-19 brain in the pyramid. Note the labeling of microglia. **(B)** Immunohistochemical stains for TREM2 in a COVID-19 brain in the ION. Note the absence of strong labeling of microglial cell bodies. Scale bars = 50 μm.

SUPPLEMENTARY FIGURE 2 C3 expression in ION astrocytes. **(A)** Immunofluorescent images of the ION labeled for nuclei (DAPI - blue) and ALDH1L1 (green) to detect astrocytes (upper row), and C3 (white – middle row). Merged panels are show on the bottom row. Arrow indicates a ALDH1L1, DAPI and C3 positive cells and arrowheads indicate C3 negative astrocyte. Scale bar = 10μm. The Condition is shown by column. **(B)** Quantification of the proportion of MT3 positive astrocytes in the different ION regions. N= 17 for COVID-19 MN and 10 for ARDS controls. Data is shown as mean +/- SEM. P value= dorsal: 0.8465, lateral: 0.7734 and ventral: 0.4666.

SUPPLEMENTARY FIGURE 3 MT3 expression in ION astrocytes. **(A)** Immunofluorescent images of the ION labeled for nuclei (DAPI - blue) and ALDH1L1 (green) to detect astrocytes (upper row), and MT3 (white – middle row). Merged panels are show on the bottom row. Arrow indicates a ALDH1L1, DAPI and MT3 positive cells and arrowheads indicate MT3 negative astrocyte. Scale bar = 10μm. **(B)** Quantification of the proportion of MT3 positive astrocytes in the different ION regions. One way BrownForsythe and Welch ANOVA correcting for multiple comparisons using Original FDR method of Benjiamini and Hochberg. Comparisons are against ARDS. *N*= 10 for COVID19-MN, 8 for COVID-19 No-MN, 10 for ARDS. *P* values =0.0120 for COVID-19 MN 0.5410 for No-MN in the lateral ION. Data is shown as mean +/- SEM. MN: COVID19 with microglial nodules. No-MN: COVID-19 with no microglial nodules.

SUPPLEMENTARY FIGURE 4 CD44 and CLU expression in ION astrocytes. **(A)** Immunofluorescent images of the ION labeled for nuclei (DAPI - blue) and GFAP (green) to detect astrocytes (upper row), and CD44 (white – second row), and CLU (third row – red). Merged CLU GFAP panels are show on the bottom row. Scale bar = 20 μm. **(B)** Quantification of CD44 positive area in the different ION regions. **(C)** Quantification of CLU positive astrocytes in the different ION regions. Unpaired two-tailed t-test. *N*= 10 for COVID-19, 18 for ARDS. *P* values = dorsal: 0.0790, lateral: 0.4304 and ventral: 0.2036. Data is shown as mean +/- SEM.

SUPPLEMENTARY FIGURE 5 Contributions of data points to principal components and hierarchical clusters. **(A)** PCA plot depicting the extent to which each immunohistochemical variable is responsible for variation in dimensions 1 and 2. **(B)** Correlation plot with meta data variables along the y-axes and dimensions from PCA analysis on the x-axis. Size and color of each dot represent the extent to which each variable’s cos2 value from PCA is represented in each dimension. **(C)** Heatmap with the numbers of samples (images) versus metadata variables in each hierarchical cluster. Rows represent each of the four hierarchical clusters.
