# Supplementary figures and images for: The link between SARS-CoV-2 related microglial reactivity and astrocyte pathology in the inferior olivary nucleus
Source: Front Neurosci. 2023 Jun 28;17:1198219. doi: 10.3389/fnins.2023.1198219 (PMC10359900; doi:10.3389/fnins.2023.1198219)

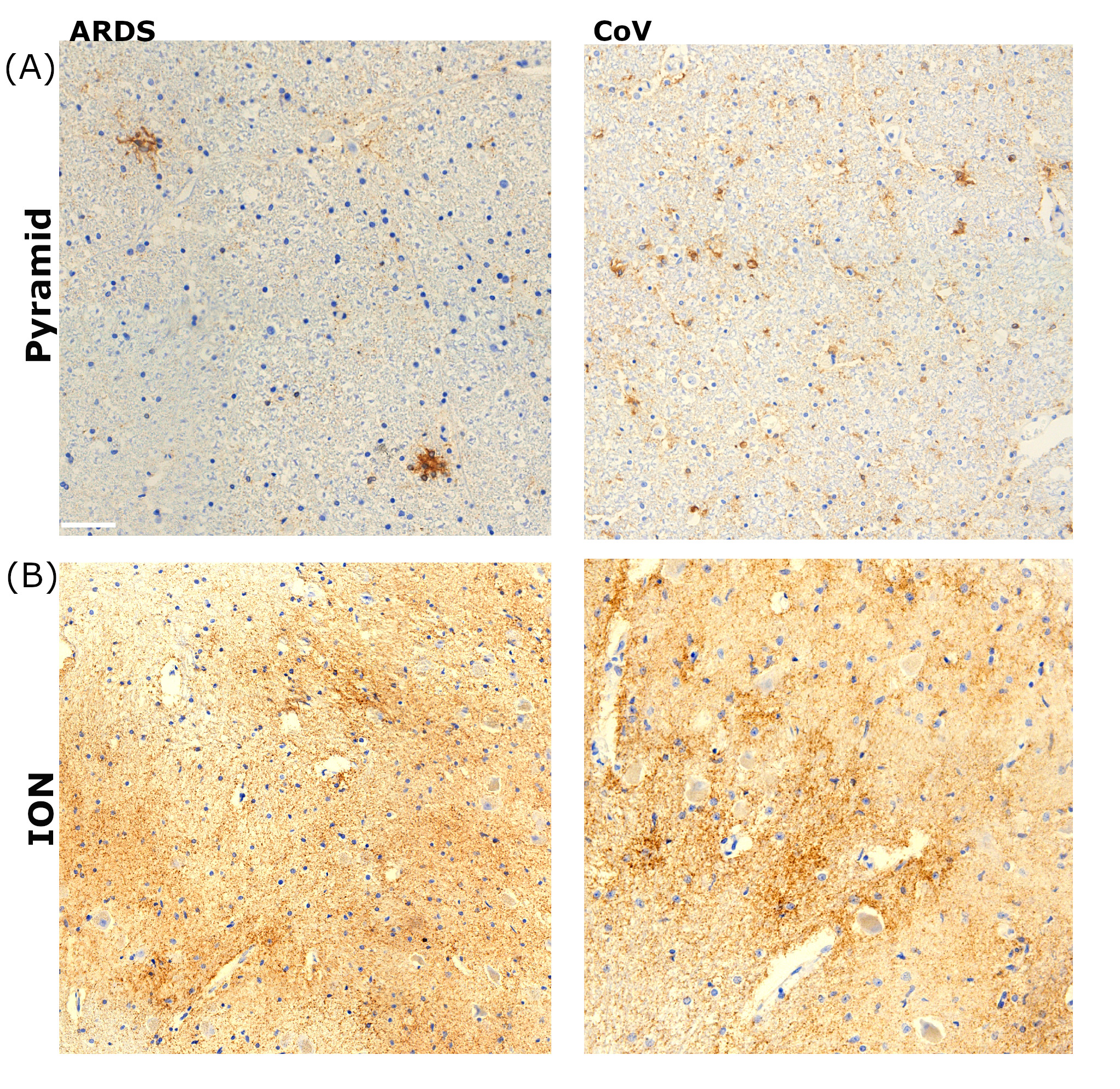

Supplement: SUPPLEMENTARY FIGURE 1 — TREM2 expression in ION microglia. (A) Immunohistochemical stains for TREM2 in a COVID-19 brain in the pyramid. Note the labeling of microglia. (B) Immunohistochemical stains for TREM2 in a COVID-19 brain in the ION. Note the absence of strong labeling of microglial cell bodies. Scale bars = 50 μm. [file Figure_1.jpeg]

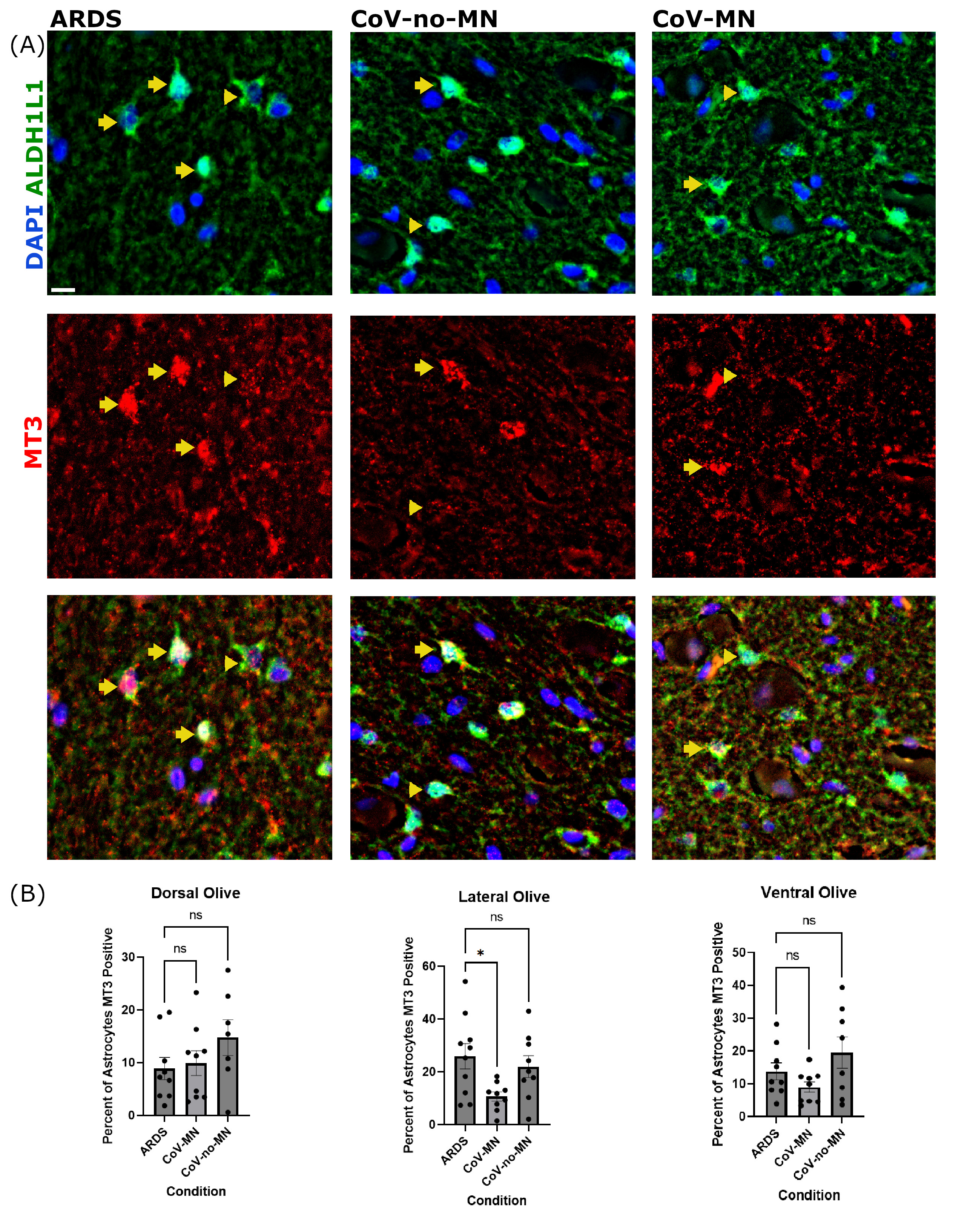

Supplement: SUPPLEMENTARY FIGURE 2 — C3 expression in ION astrocytes. (A) Immunofluorescent images of the ION labeled for nuclei (DAPI - blue) and ALDH1L1 (green) to detect astrocytes (upper row), and C3 (white – middle row). Merged panels are show on the bottom row. Arrow indicates a ALDH1L1, DAPI and C3 positive cells and arrowheads indicate C3 negative astrocyte. Scale bar = 10μm. The Condition is shown by column. (B) Quantification of the proportion of MT3 positive astrocytes in the different ION regions. N= 17 for COVID-19 MN and 10 for ARDS controls. Data is shown as mean +/- SEM. P value= dorsal: 0.8465, lateral: 0.7734 and ventral: 0.4666. [file Figure_2.jpeg]

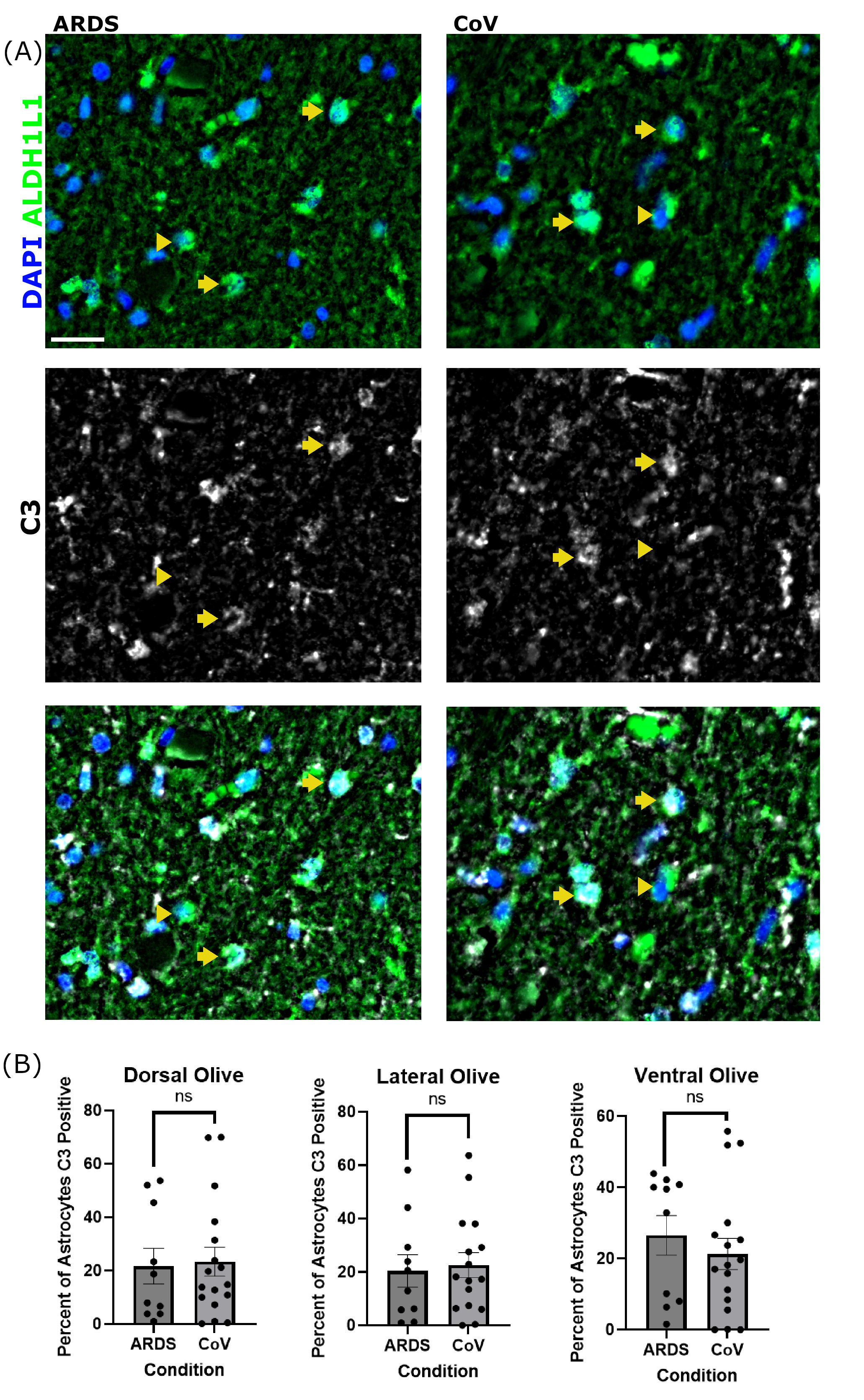

Supplement: SUPPLEMENTARY FIGURE 3 — MT3 expression in ION astrocytes. (A) Immunofluorescent images of the ION labeled for nuclei (DAPI - blue) and ALDH1L1 (green) to detect astrocytes (upper row), and MT3 (white – middle row). Merged panels are show on the bottom row. Arrow indicates a ALDH1L1, DAPI and MT3 positive cells and arrowheads indicate MT3 negative astrocyte. Scale bar = 10μm. (B) Quantification of the proportion of MT3 positive astrocytes in the different ION regions. One way BrownForsythe and Welch ANOVA correcting for multiple comparisons using Original FDR method of Benjiamini and Hochberg. Comparisons are against ARDS. N= 10 for COVID19-MN, 8 for COVID-19 No-MN, 10 for ARDS. P values =0.0120 for COVID-19 MN 0.5410 for No-MN in the lateral ION. Data is shown as mean +/- SEM. MN: COVID19 with microglial nodules. No-MN: COVID-19 with no microglial nodules. [file Figure_3.jpeg]

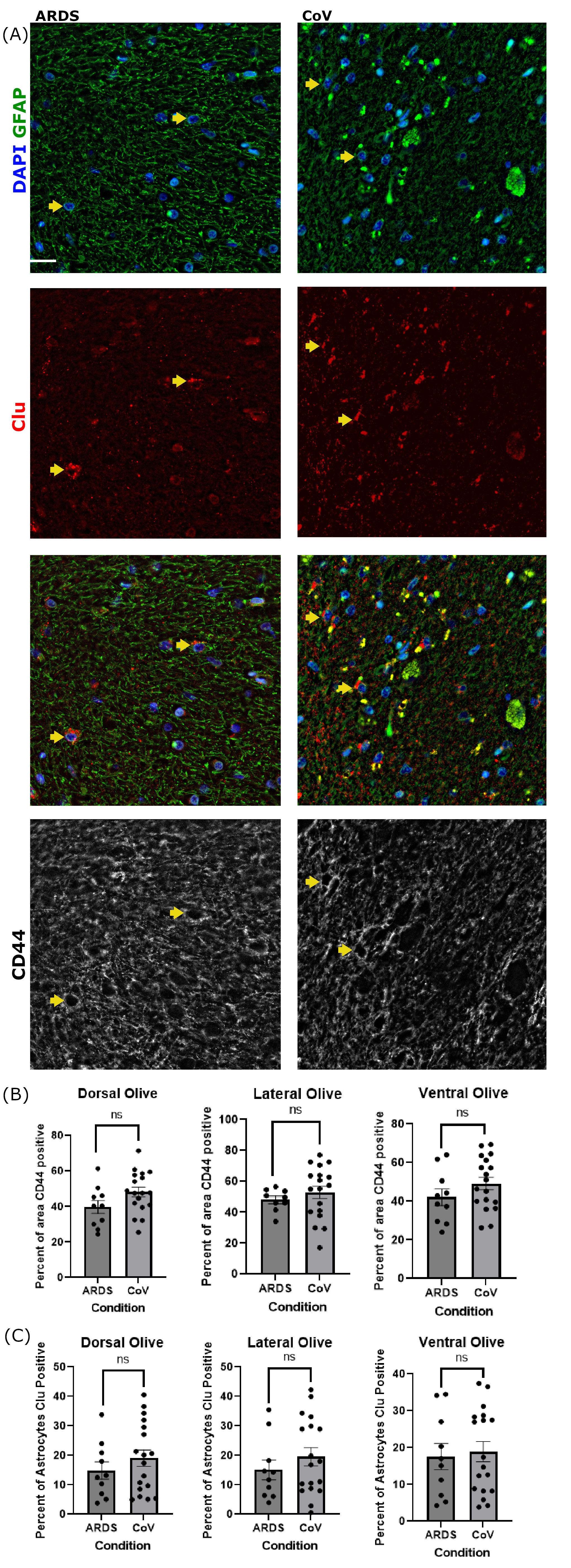

Supplement: SUPPLEMENTARY FIGURE 4 — CD44 and CLU expression in ION astrocytes. (A) Immunofluorescent images of the ION labeled for nuclei (DAPI - blue) and GFAP (green) to detect astrocytes (upper row), and CD44 (white – second row), and CLU (third row – red). Merged CLU GFAP panels are show on the bottom row. Scale bar = 20 μm. (B) Quantification of CD44 positive area in the different ION regions. (C) Quantification of CLU positive astrocytes in the different ION regions. Unpaired two-tailed t-test. N= 10 for COVID-19, 18 for ARDS. P values = dorsal: 0.0790, lateral: 0.4304 and ventral: 0.2036. Data is shown as mean +/- SEM. [file Figure_4.jpeg]

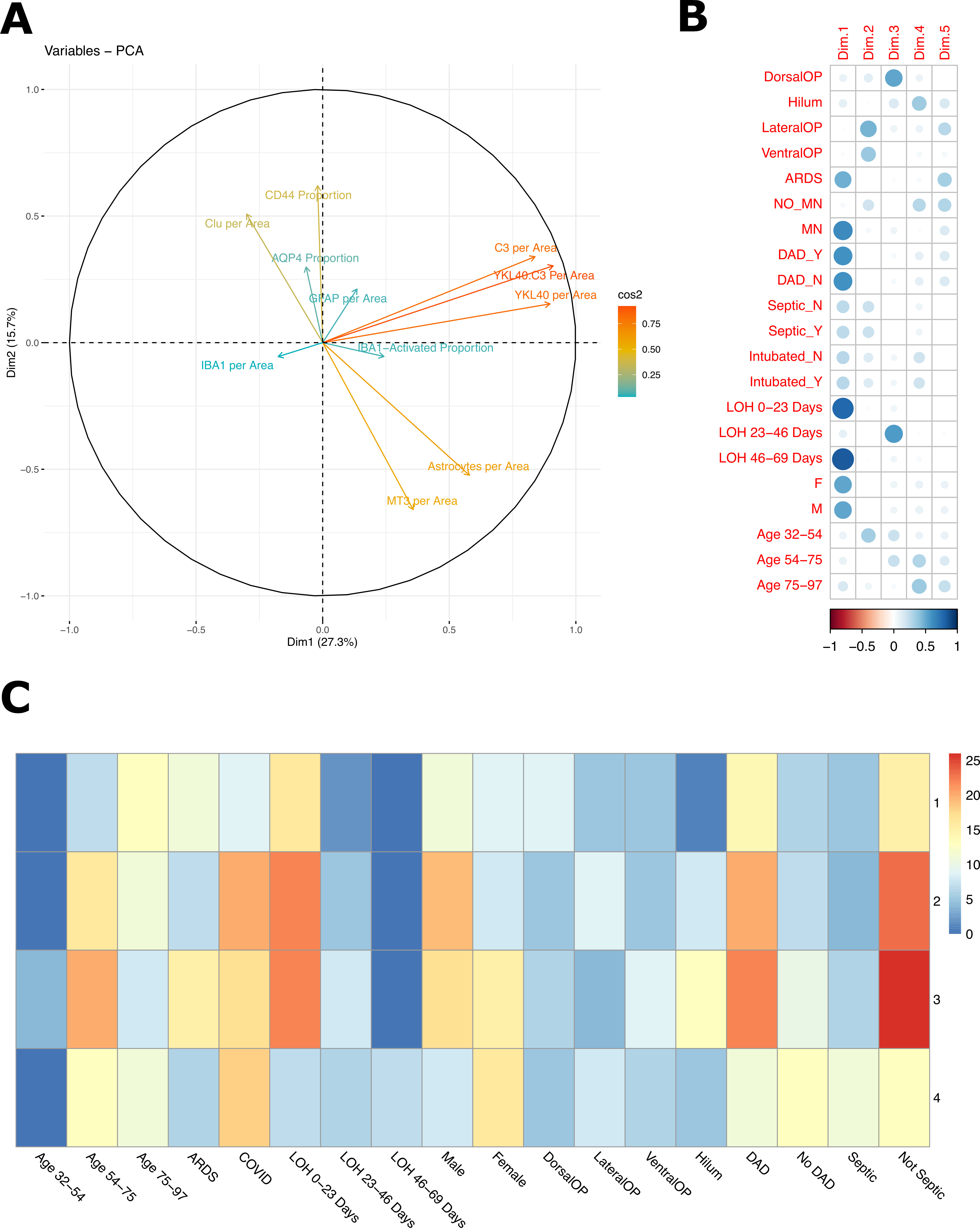

Supplement: SUPPLEMENTARY FIGURE 5 — Contributions of data points to principal components and hierarchical clusters. (A) PCA plot depicting the extent to which each immunohistochemical variable is responsible for variation in dimensions 1 and 2. (B) Correlation plot with meta data variables along the y-axes and dimensions from PCA analysis on the x-axis. Size and color of each dot represent the extent to which each variable’s cos2 value from PCA is represented in each dimension. (C) Heatmap with the numbers of samples (images) versus metadata variables in each hierarchical cluster. Rows represent each of the four hierarchical clusters. [file Figure_5.jpeg]
